# Induced Second Trimester Abortion and Associated Factors in Amhara Region Referral Hospitals

**DOI:** 10.1155/2015/256534

**Published:** 2015-03-30

**Authors:** Amlaku Mulat, Hinsermu Bayu, Habtamu Mellie, Amare Alemu

**Affiliations:** ^1^Department of Midwifery, College of Health Sciences, Mekelle University, P.O. Box 1871, Mekelle, Ethiopia; ^2^Department of Public Health, College of Medicine and Health Sciences, Debre Markos University, Debre Markos, Ethiopia; ^3^Department of Midwifery, College of Medicine and Health Sciences, Bahirdar University, Bahirdar, Ethiopia

## Abstract

*Background*. Although the vast majority of abortions are performed in the first trimester, still 10–15% of terminations of pregnancies have taken place in the second trimester period globally. As compared to first trimester, second trimester abortions are disproportionately contribute for maternal morbidity and mortality especially in low-resource countries where access to safe second trimester abortion is limited. The main aim of this study was to assess the prevalence and associated factors of induced second trimester abortion in Amhara region referral hospitals, northwest Ethiopia. *Methods*. Institution based cross-sectional study was conducted in Amhara region referral hospitals among 416 women who sought abortion services. Participants were selected using systematic sampling technique. Data were collected using pretested structured questionnaire through interviewing. After the data were entered and analyzed; variables which have *P* value < 0.2 in bivariate analysis, not colinear, were entered into multiple logistic regressions to see the net effect with 95% CI and *P* value < 0.05. *Results*. The prevalence of induced second trimester abortion was 19.2%. Being rural (AOR = 1.86 [95% CI = 1.11–3.14]), having irregular menstrual cycle (AOR = 1.76 [95% CI = 1.03–2.98]), not recognizing their pregnancy at early time (AOR = 2.05 [95% CI = 1.21–3.48]), and having logistics related problems (AOR = 2.37 [95% CI = 1.02–5.53]) were found to have statistically significant association with induced second trimester abortion. *Conclusion*. Induced second trimester abortion is high despite the availability of first trimester abortion services. Therefore, increase accessibility and availability of safe second trimester abortion services below referral level, counseling and logistical support are helpful to minimize late abortions.

## 1. Introduction

Second trimester abortion is termination of pregnancy in a period from 13 to 28 weeks of gestation, which again is subdivided into early period between 13 and 20 weeks and late period between 20 and 28 weeks [1]. Globally, over 42 million abortions are performed annually and 10–15% of the cases take place in second trimester period, over half of which are considered unsafe, and disproportionately contribute to maternal deaths [2]. In 2008, there were 29 abortions per 1,000 women aged 15–44 years in developing countries, compared with 24 per 1,000 in the developed world [3, 4].

As researches showed, the prevalence of induced second trimester abortion was as high as 25%–30% in India and South Africa [5], 8.6% in England and Wales [6], 34% in Kenya [7], 10% in Nigeria [8], and in 2008 there were 382,000 induced abortions performed in Ethiopia; about 42% of pregnancies were unintended and the rate of abortion was 101 per 1,000 women [9].

Abortion-related complications account for approximately 13% of maternal deaths worldwide, roughly estimated as 47000 deaths per year. Second trimester abortion carries a higher risk of morbidity and mortality as compared to first trimester abortion specially in developing countries [10]. In sub-Saharan Africa from overall unsafe abortion complications, 2/3 of them are attributable to the second trimester period [11]. More than one-third of all women with abortion complications were seeking care after second trimester abortion and it was more common among women who lived in rural areas than among their urban counterparts in Ethiopia [12].

The Federal Ministry of Health in 2006 estimated that abortion-related deaths accounted for more than 30% of maternal deaths in Ethiopia. Besides this, access to second trimester abortions is severely limited. Only 9–10% of all facilities have a provider who can perform this service [12].

As evidences showed, factors associated with second trimester termination of pregnancies were low use of modern contraceptive methods, restrictive abortion laws and policies, gender discrimination and lack of safe abortion services, stigma associated with abortion [11, 13–19], and relatively higher cost of late abortion care for nonmedical reasons though it was free of charge for medical reasons in countries like Mongolia [20]. Delay in making decision in first trimester due to either family/partner pressure or conflicting feeling about the pregnancy, lack of information about where to obtain an abortion, lack of providers, delay in testing and appointments, abortion associated stigma, the need to travel far from their home, and evidence of fetal anomalies or threats to health of mother were contributor to second trimester abortion [6, 21–23]. A retrospective study conducted in Israel showed that age below 18 years, age above 40 years, or pregnancy outside of marriage were forces to have second trimester abortion [24]. A cross-sectional study in Amhara region reveals that 31.3% of women aged 15 to 49 were committing induced abortion due to fear of family and the community, and 14.1% were due to economic problem [9].

The Millennium Development Goal (MDG) to improve maternal health is unlikely to be achieved without addressing unsafe abortion and associated mortality and morbidity. Different study suggests that second trimester abortion is usually associated with higher rates of complications as compared to first trimester abortion [1, 25]. Therefore, assessing the prevalence and associated factors of induced second trimester abortion is important for setting interventions to reduce the proportion of unsafe second trimester abortions and thereby saving the life of mothers from abortion-related morbidity and mortality.

## 2. Methods

### 2.1. Study Design, Area, and Period

Institution based cross-sectional study was conducted in Amhara region referral hospitals. Based on the 2007 Census, the Amhara region has a total population of 17,221,976 of whom 8,580,396 were women. The region has 5 referral hospitals, 19 general/district hospitals, 220 health centers, and 2,941 health posts. The study was conducted from July 5, 2013, to January 5, 2014 (for 6 months).

All women who came for abortion service during the study period in Amhara region referral hospitals were the source population for study. Women excluded from study were those having gestational trophoblastic disease (partial mole) and those who cannot hear or are seriously ill with coma during data collection period.

### 2.2. Sampling

The sample size was calculated using single population proportion formula with 50% prevalence of second trimester abortion due to no previous study. Assuming a marginal error of 5% and 10% nonrespondent rate, the estimated sample size was 422.

### 2.3. Sampling Procedures

The sample for each referral hospital was arranged based on their patient flow by reviewing the 6-month report of the previous year. After proportional allocation of the samples for each referral hospital, systematic sampling technique was used to select the study subjects and participants were interviewed based on their exit after they received all the necessary abortion care.

### 2.4. Data Collection Procedures and Statistical Analysis

Structured questionnaire which was developed by reviewing different literatures was used for the study. The questionnaire was prepared in English, translated to Amharic, and then translated back to English to check for consistency. Data was collected via exit interview. The data was collected by five BSc degree holder midwives, one in each referral hospital. Two supervisors who have BSc degree in midwifery were assigned for supervisory activities along with the principal investigator. Training was given to the data collectors and supervisors on the objective, relevance of the study, confidentiality of information, respondent's right, informed consent, and techniques of interview.

Before the actual data collection, pretest was conducted in Finote Selam Zonal Hospital for one month with 21 clients in May 2013 to ensure the validity of the survey tool and to standardize the questionnaire. The supervisor and the principal investigator made frequent checks on the data collection process to ensure the completeness and consistency of the gathered information and errors found during the process were also corrected.

Induced second trimester abortion was the dependent variable for study and the independent variables were sociodemographic factors (age, marital status, educational status, residence, monthly income, religion, ethnicity, husband's occupation, and educational status), reproductive characteristics (nature of menses, gravidity, parity, number of live births, current conditions of pregnancy, contraceptive history, and abortion history), logistical factors (taking time while finding money, transportation problem, referral problem, and not having information about where the service is given, distant from the institute), and medical factors (fetal deformity, maternal illness).

After data were collected, each questionnaire was given code and checked visually for completeness. The data were entered into Epi-info version 3.5.1 and transported to SPSS version 20 software packages for analysis. Data cleaning was performed using frequencies, sorting, listing to see missed values, and outliers and then correction was made by reviewing the original paper. Bivariate analysis was carried out first to observe the crude association between independent and outcome variables. The variables which have *P* value < 0.2 in bivariate analysis, not colinear, were entered into multiple logistic regressions to assess the net effect by controlling confounders. Finally, statistically significant variables which fit final regression model were identified using odds ratio with 95% confidence interval and *P* value < 0.05.

## 3. Ethical Consideration

Ethical clearance was obtained from Ethical Review Board (ERB) of University of Gondar Department of Midwifery. Letter of cooperation was obtained from the Amhara Regional Health Bureau and submitted to each referral hospital. Informed consent was also obtained from each client and introduced the objective of the study that it contributes to set interventions and strategies to improve services. Any client who was not willing to participate in the study had the right to refuse at any time of interviewing. Data were collected after full informed written consent is obtained and confidentiality of the information was maintained by excluding names as identification in the questionnaire and keeping their privacy during the interview by interviewing them alone.

## 4. Results

Out of 422 women who sought abortion services in Amhara region referral hospitals 416 women completed the interview administer questionnaire. Therefore, data analysis was made based on 416 cases that have been completing the interview. Response rate was 98.6%.

### 4.1. Sociodemographic Characteristics

Among study participants more than one-third (141) (33.6%) of them were in the age range of 20 and 24 years and the mean age was 24.4 (±6.32). Majority of the respondents were urban by residence (252) (60.6%), Orthodox Christian (318) (76.4%), married (193) (46.4%), and housewives (154) (37%), and not able to read and write (124) (29.8%) and are having monthly income of <26.3 dollars (323) (77.6%) and the median monthly income was 18.4 dollars. Among married women, their husbands' occupations are as follows: 88 (21.2%) were private employee, 50 (12%) government employee, 39 (9.4%) farmer, and 16 (3.8%) unemployed. Regarding husband's educational status of married women 56 (13.5%) were able to read and write, 50 (12%) were unable to read and write, 43 (10.3%) had diploma and above, 29 (7%) 9–12th, and 15 (3.6%) 1–8th (Table 1).

### 4.2. Reproductive Characteristics

Two hundred sixty-eight (64.4%) of respondents had regular menstrual cycle and the remaining one hundred forty-eight (35.6%) had irregular menstrual cycle prior to the current abortion. Nearly sixty percent of the respondents were pregnant for the first time (248) (59.6%), even though the pregnancy ended up with abortion. Majority of the respondents (333) (80%) had been pregnant in the range between 1 and 3 times and the mode of gravidity was 1. More than half of the cases (220) (52.9%) were nullipara. Two hundred thirty-two (55.8%) of the respondents did not have live birth (Table 2).

Only fifty-five (13.2%) of women had abortion previously from which 38 (9.1%) of them had termination of pregnancy at health institution and the remaining 17 (4.1%) were spontaneously aborted at their home. From those who had abortion previously, 53 (12.7%) of them had abortion 1 or 2 times and only 2 (0.5%) had abortion more than 3 times.

About 248 (59.6%) of the respondents did not plan their pregnancies. However, terminated due to different nonmedical reasons such that 116 (27.9%) of women did not want to lose their job/drop out of school as a result of the pregnancy, 88 (21.2%) of them could not afford to cater a baby, 15 (3.6%) had unsupportive friend/husband, and 22 (5.3%) wanted to increase birth interval and only 7 (1.7%) of terminations of pregnancy were because the woman is too young to raise a child.

On the other hand, one hundred sixty-eight (40.4%) pregnancies were planned but terminated due to medical reasons such as 132 (31.7%) due to the problem of the fetus and 25 (6%) as a result of chronic illness of the mother and only 11 (2.6%) of them were terminated because the continuation of pregnancy threatens the life of the women.

### 4.3. Prevalence of Induced Second Trimester Abortion

During the study period out of 416 women who sought abortion service in Amhara region referral hospitals 80 (19.2%) of respondents had induced abortion and 60 (14.4%) of respondents had spontaneous abortion in the second trimester period.

**Figure 1:**
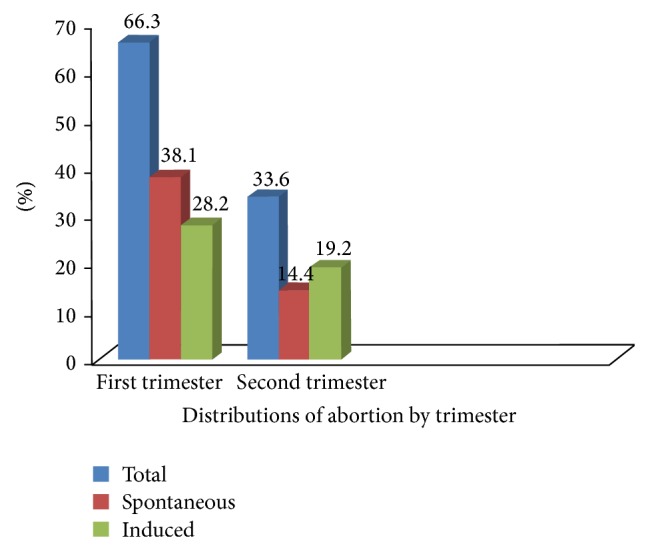
Distribution of abortion by trimester in Amhara region referral hospitals.

Seventy-six (18.2%) of respondents had induced abortion during the early second trimester period between the gestational age of 13 and 20 weeks whereas the remaining four (1%) were terminated between 21 and 28 weeks during late second trimester period. However, majority 276 (66.3%) of respondents had abortion in the first trimester period (Figure 1).

### 4.4. Reasons for Delayed Seeking of Abortion Care

Nearly three-fourths of the respondents (299) (71.9%) took ≤10 days from confirming their pregnancy until they decided to have abortion, some of them (72) (17.3%) took from 11 days up to 20 days, and only 45 (10.8%) of the cases last 21 days and above.

Women who did not plan their pregnancy had mentioned different reasons why they did not seek early abortion service in the first trimester period. Out of 248 (59.6%) unplanned pregnancies which were terminated due to different reasons, 186 (44.7%) of the respondents were late because they did not recognize they were pregnant, 22 (5.3%) took time while discussing about abortion with family/husbands, 17 (4.1%) were confused either to terminate or to continue by peer pressure, 13 (3.1%) took time because they were afraid to tell their family, and 10 (2.4%) did not expect relationship with their husbands/freinds are changed.

### 4.5. Logistical Problems

Three hundred twenty-two (77.4%) of the women who had abortion in the specified referral hospitals faced different problems related to logistics. About 178 (42.8%) of the respondents waste their time while they found money for abortion expense, 96 (23.1%) did not have information about where the abortion service is given, 23 (5.5%) due transportation delay, 18 (4.3%) due to referral delay from health center and only 7 (1.7%) mentioned as they were distant from the institute.

### 4.6. Associated Factors of Induced Second Trimester Abortion

The residence of women, nature of menses, number of pregnancies, getting off from work, not being able to afford to cater a baby, not recognizing their pregnancy, and problem related to logistics were found to be associated with second trimester abortion in the bivariate analysis. Those variables were entered once into a backward stepwise multiple logistic regression model. Finally the model retains only those factors with significant associations at *P* value < 0.05 level. After adjustment, factors that increase risk of second trimester abortion were being more rural than urban by 1.86 times, having irregular menstrual cycle compared to regular by 1.76 times, not recognizing their pregnancy at early time compared to their counterparts by 2.05 times, and having logistics related problems (spent much time finding money for abortion expense) compared to not having problems by 2.37 times (Table 3).

## 5. Discussion

This study demonstrated that the overall prevalence of induced second trimester abortion was 19.2% which is in line with the prior study in South Africa that was 20% [26]. However, the finding of this study is greater than the global reported proportion 10–15% [10], study done in Nigeria 10% [8] and New Delhi 2% [27]. The possible explanation might be due to many women presenting to the government hospitals because private health institutions are not equipped to provide abortion care beyond 12 weeks of gestation except in rare occasions like private hospitals staffed by specialists (obstetrician/gynecologist). Moreover, different delaying factors may affect the women not to seek early abortion care in the first trimester period.

More than one-third of all women with abortion complications passed first trimester period and seeking care after second trimester abortion was more common among women who lived in the countryside as compared to urban residence in Ethiopia [12]. Likewise this study also revealed that being from rural residence is 1.86 times more likely to have abortion in the latter half of second trimester period as compared to urban women (AOR = 1.86 [95% CI = 1.11–3.14]). Possibly women from the countryside may not have information about where the appropriate service is given and usually they are far from the institution.

A study done in New Delhi showed that factors affecting having second trimester abortion are difficulty in recognition of pregnancy and delay related to logistic problems [27].

The present study also highlighted that those women who did not recognize their pregnancy early were two times more likely to miss the opportunity to have abortion in the first trimester period than their counterparts (AOR = 2.05 [95% CI = 1.21–3.48]). The possible explanation might be irregular nature of women's menses results unsure of the missing period, thereby unable to pick early symptoms of pregnancy, and still time is needed for arrangements to have pregnancy test.

The strength of current study might be coverage of all referral hospitals within the region and relatively long duration of data collection over 6 months which could increase the representativeness of samples that possibly predict the outcome variable. However, this study might have limitations like any other cross-sectional study. It could not explain cause and effect relationship, and some respondents might not correctly remember their menstrual cycle prior to pregnancy.

In conclusion, the prevalence of induced second trimester abortion is high despite the availability of safe first trimester abortion services. Different delaying factors were preventing the women from getting early abortion services. Women who had faced problems related to logistics, women from rural residence, women who are unable to recognize their pregnancy early, and irregular nature of their menses were found to have an abortion in the second trimester period. Therefore Amhara Regional Health Bureau should further expand and strengthen safe second trimester abortion services below referral hospitals level to the level of general and district hospitals and make sure that accessible and affordable services are given. Organizations working on maternal health at different levels should give counseling for mothers to early recognize their pregnancy and to make decisions regarding either to continue it or not as early as possible. In addition, they should give logistical support for mothers who want to have abortion.

Further qualitative investigation is needed on underlying factors why women make late decision to terminate their pregnancy in the second trimester period.

## Figures and Tables

**Table 1:** Sociodemographic characteristics of women who sought abortion service in Amhara region referral hospitals.

Variables	Frequency (*n* = 416)	Percent
Age		
15–19	101	24.3
20–24	141	33.9
25–29	78	18.8
30–34	53	12.7
≥35	43	10.3
Residence		
Urban	252	60.6
Rural	164	39.4
Ethnicity		
Amhara	389	93.5
Oromo	7	1.7
Tigre	14	3.4
Others^*^	6	1.4
Religion		
Orthodox	318	76.4
Muslim	91	21.9
Protestant	4	1.0
Catholic	3	0.7
Marital status		
Single	173	41.6
Married	193	46.4
Divorced	44	10.6
Widowed	6	1.4
Occupation		
Housewife	154	37.0
Student	110	26.4
Merchant	44	10.6
Government employee	44	10.6
Farmer	12	2.9
Daily laborer	36	8.7
Others^**^	16	3.8
Monthly income		
<26.3 dollars	323	77.6
26.3–52.6 dollars	65	15.6
>52.6 dollars	28	6.8
Educational status		
Unable to read and write	124	29.8
Able to read and write	45	10.8
1–8th	98	23.6
9–12th	110	26.4
Diploma and above	39	9.4

*Note*: others^*^  =  Agew, Afar, others^**^ =  housemaid, jobless.

**Table 2:** Reproductive characteristics of women who sought abortion service in Amhara region referral hospitals (*n* = 416).

Variables	Frequency	Percent
Nature of menses		
Regular	268	64.4
Irregular	148	35.6
Got pregnant before		
It is my first time	248	59.6
Pregnant before	168	40.4
Gravidity		
1–3	333	80.0
4–6	60	14.4
≥7	23	5.6
Parity		
0	220	52.9
1	69	16.6
2	52	12.5
≥3	75	18.0
Number of live births		
0	232	55.7
1	74	17.8
2	49	11.8
≥3	61	14.7

**Table 3:** The bivariate and multiple logistic regression results on factors associated with induced second trimester abortion.

Characteristics	Induced second trimester abortion			
	No	Yes	With 95% CI	
	Frequency (%)	Frequency (%)	COR	AOR
Residence				
Urban	215 (85.3)	37 (14.7)	1	1
Rural	121 (73.8)	43 (26.2)	2.06 (1.26–3.38)	**1.86 (1.11–3.14)** ^**^
Number of pregnancies				
First *p*_*x*_	191 (77)	57 (23)	1	1
2nd *p*_*x*_ and above	145 (86.3)	23 (13.7)	0.53 (0.31–0.90)	0.79 (0.45–10.43)
Nature of menses				
Regular	231 (86.2)	37 (13.8)	1	1
Irregular	105 (70.9)	43 (29.1)	2.56 (1.56–4.20)	**1.76 (1.03–2.98)** ^*^
Get off from work				
No	250 (83.3)	50 (16.7)	1	1
Yes	86 (74.1)	30 (25.9)	1.74 (1.04–2.92)	1.19 (0.62–2.29)
Did not know she was pregnant				
No	200 (87)	30 (13)	1	1
Yes	136 (73.1)	50 (26.9)	2.45 (1.48–4.05)	**2.05 (1.21–3.48)** ^*^
Cannot afford to cater baby				
No	272 (82.9)	56 (17.1)	1	1
Yes	64 (72.7)	24 (27.3)	1.82 (1.05–3.16)	1.31 (0.71–2.39)
Logistical problem				
No	87 (92.6)	7 (7.4)	1	1
Yes	249 (77.3)	73 (22.7)	3.64 (1.62–8.22)	**2.37 (1.02–5.53)** ^*^

*Note*; *p*_*x*_ = pregnancy, COR = crude odds ratio, and AOR = adjusted odds ratio; ^*^ *P* < 0.05 and ^**^ *P* < 0.001.
